# Classical pellagra, the disease of 4 Ds, the forgotten entity

**DOI:** 10.11604/pamj.2020.36.219.24806

**Published:** 2020-07-27

**Authors:** Vikash Paudel, Deepa Chudal

**Affiliations:** 1 National Medical College, Birgunj, Parsa, Nepal,; 2 Nepal Police Hospital, Kathmandu, Nepal

**Keywords:** Niacin, pellagra, poverty

## Image in medicine

Pellagra is a nutritional disorder due to deficiency of vitamin niacin (vitamin-B3) or its precursor tryptophan, which was first described by a Spanish physician, Gaspar Casal (1735AD). Classically, it manifests with triad of diarrhea, dermatitis and dementia and if not treated in time leading to death. Thus, it’s a disorder affecting the skin, nervous system, and gastrointestinal. It is commonly associated with malnutrition and alcoholism. It’s mostly found in some areas of India, China, and Africa where corn is used as a staple food and poverty supervenes. We present a case of a middle-aged male clinically diagnosed to 3Ds of pellagra and successfully treated with vitamin B3 supplementation to prevent the fourth D i.e. death. A forty-two-year farmer from hilly area of central Nepal, presented with pruritic and eczematous skin lesions over dorsum of hands and forearms, face, neck (A) and lower leg with mild confusion state and diarrhea of 2 months duration. There was no history of fever, seizures or jerky movements or weakness of limbs. His family was on stable diet of maize and was also an occasional alcohol consumer. Laboratory investigation revealed mild anemia and serum niacin level was found to be low. The diagnosis of a classical pellagra was established. We supplemented with combination of vitamin B complex with higher dose of niacin with abstaining him from alcohol and providing niacin rich diet. The condition improved within a period of two weeks and two months of follow up, most of his signs and symptoms were subsided (B).

**Figure 1:**
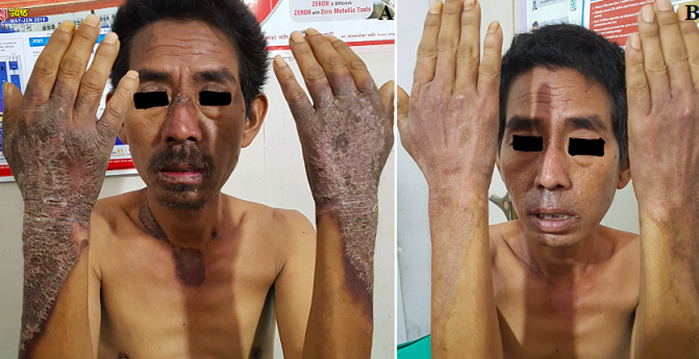
classical skin lesions in pellagra involving face, neck, hand and forearm (A), after treatment with niacin for 2 weeks (B)

